# The Impact of Dialysis Duration on Multidimensional Health Outcomes: A Cross-Sectional Study

**DOI:** 10.3390/jcm14020376

**Published:** 2025-01-09

**Authors:** Leszek Sułkowski, Andrzej Matyja, Maciej Matyja

**Affiliations:** 1 Department of General Surgery, Regional Specialist Hospital, 42-218 Częstochowa, Poland; leszeksulkowski@szpitalparkitka.com.pl; 2 2nd Department of General Surgery, Jagiellonian University Medical College, 30-688 Kraków, Poland

**Keywords:** dialysis, modified fatigue impact scale (MFIS), pain effect scale (PES), sexual satisfaction scale (SSS), bowel control scale (BCS), impact of visual impairment scale (IVIS), perceived deficits questionnaire (PDQ), mental health inventory MHI), modified social support survey (MSSS), the World Health Organization quality of life questionnaire-bref version (WHOQOL-Bref), cantill ladder

## Abstract

**Background:** Dialysis patients face multidimensional challenges that affect their quality of life. This study aimed to evaluate the association between dialysis duration and various physical, cognitive, and psychosocial parameters, including fatigue, pain, sexual satisfaction, bowel control, vision, cognitive deficits, mental health, social support, quality of life, and life satisfaction, while incorporating sociodemographic data for greater context. **Methods:** A cross-sectional study was conducted using validated instruments such as the Modified Fatigue Impact Scale (MFIS), Pain Effect Scale (PES), Sexual Satisfaction Scale (SSS), Bowel Control Scale (BWCS), Impact of Visual Impairment Scale (IVIS), Perceived Deficits Questionnaire (PDQ), Mental Health Inventory (MHI), Modified Social Support Survey (MSSS), WHOQOL-BREF, and Cantril Ladder. Associations between dialysis duration and these parameters were analyzed. Correlations between current and future life satisfaction were also examined. **Results:** Dialysis duration significantly affected sexual satisfaction, with scores worsening over time (*p* = 0.029). Cognitive deficits in planning and organization exhibited a near-significant trend (*p* = 0.072). Patients with low current life satisfaction anticipated significant future declines (*p* = 0.001). However, no significant associations were observed between dialysis duration and fatigue, pain, bowel control, vision, mental health, social support, overall quality of life, or life satisfaction. **Conclusions:** Prolonged dialysis negatively influences sexual satisfaction and may impact specific cognitive domains. The relationship between current and expected life satisfaction highlights the importance of addressing psychological health in this population. While other parameters remained unaffected, individualized care strategies focusing on sexual, cognitive, and psychological support could improve outcomes. Future research should focus on exploring these relationships further and developing targeted interventions to address vulnerable areas, such as sexual, cognitive, and psychological health.

## 1. Introduction

Patients’ lives extend beyond the confines of their disease, encompassing physical, psychological, and social dimensions that collectively shape their quality of life [1]. To genuinely improve outcomes for patients, it is essential to address not only their physical functioning but also their emotional and social well-being [1]. The subjective feelings and thoughts patients harbor about their disease significantly influence health-related quality of life, which is notably impaired in individuals with end-stage renal disease [1]. Although factors such as comorbid conditions, hemoglobin levels, and residual renal function explain poor quality of life to some extent, these factors are insufficient to capture the full picture [2]. Merkus et al. emphasize the importance of integrating quality of life metrics into the routine monitoring of dialysis patients [2].

The global prevalence of end-stage renal disease is increasing, with older individuals, particularly those aged 65 and above, representing a growing segment of the population requiring dialysis [3]. The rise in kidney failure is largely driven by the increasing prevalence of diabetes and hypertension [3]. Individuals on dialysis consistently report lower health-related quality of life compared to the general population, making this a critical area of concern [4]. End-stage renal disease imposes significant physical and psychological burdens, often coupled with a poor prognosis [5]. Cognitive impairment is frequently observed among patients with end-stage renal disease, with a multifactorial etiology that includes vascular changes, uremia, and inflammation [6]. Despite the high prevalence of cognitive dysfunction, depression, and anxiety in this population, these issues often remain undiagnosed and untreated [6]. Early intervention in addressing cognitive and psychological issues has the potential to improve quality of life substantially [6].

Fatigue is a pervasive yet under-recognized symptom among dialysis patients, with profound effects on daily functioning and mental health [7,8]. Hemodialysis patients commonly experience fatigue due to generalized weakness, muscle atrophy, and exercise intolerance [7,8]. Pain, another underappreciated yet prevalent symptom, significantly impacts individuals with end-stage renal disease, often arising from complications, comorbidities, and the dialysis process itself [9,10]. Chronic pain, particularly with neuropathic components, frequently coexists with mood disorders, sleep disturbances, and diminished life satisfaction [9].

Bowel dysfunction is another challenging issue, with many patients reporting ineffective or unacceptable management strategies for their symptoms [11]. Visual problems are similarly common, contributing to reduced functional capacity and quality of life [12,13]. Visual impairment not only hinders daily tasks but also predicts adverse clinical outcomes, including cardiovascular events and mortality in dialysis patients [12,13]. Emotional distress and limited social support further compound the challenges faced by this population, highlighting the importance of addressing psychological resilience and social interaction [1,14,15]. Limited access to social support negatively affects disease management and psychosocial functioning, underscoring its vital role in patient outcomes [1,15].

Despite substantial research on the health outcomes of dialysis patients, existing studies often focus on isolated domains such as fatigue, pain, or overall quality of life. Few studies comprehensively integrate multiple health dimensions, including psychosocial and cognitive parameters, in the context of dialysis duration. This study seeks to address these gaps by providing a multidimensional analysis of physical, cognitive, and psychosocial outcomes. The novelty of this research lies in its holistic approach, its focus on underexamined parameters, and its exploration of how these outcomes evolve with prolonged dialysis. Additionally, the relationship between current and anticipated life satisfaction over a five-year period was assessed. By doing so, we aim to provide actionable insights into the psychosocial dimensions of living with end-stage renal disease and strategies for personalized care in this vulnerable population.

## 2. Materials and Methods

The study was approved by the Bioethics Committee (approval no. K.B.Cz. 0014/2017). The patients were recruited from the dialysis Department of the Regional Specialist Hospital in Częstochowa, Poland. All participants were informed about the nature of the study, and written consent was obtained for their participation. The inclusion criteria for the study were: chronic kidney failure treated with hemodialysis, willingness to participate, providing written informed consent, and age above 18 years. Exclusion criteria were: acute kidney failure, peritoneal dialysis, age below 18 years, refusal to participate, and incomplete questionnaire responses. Patients completed the questionnaires independently, with no other person present but with a trained interviewer available to answer any questions that might arise. Patients were instructed to take into account the past 4 weeks.

To achieve the objectives of the study, patients were provided with the following diagnostic questionnaires (Table 1):

Modified Fatigue Impact Scale (MFIS): a 21-item multidimensional scale developed to assess the perceived impact of fatigue on various daily activities. It includes three subscales: Physical, Cognitive, and Psychosocial, along with a total score. Each item is designed to be self-explanatory, and higher scores indicate a greater impact of fatigue on the patient’s daily functioning. The MFIS can be used to assess the overall impact of fatigue and can also be broken down into the three subscales for a more detailed analysis. Additionally, a shortened five-item version of the MFIS (MFIS-5) is available, consisting of the five items most strongly correlated with the total MFIS score, representing all three subscales. The MFIS-5 ranges from 0 to 20. While the five-item version is useful in time-limited situations, the 21-item version is preferred for a more comprehensive evaluation, as it provides a more detailed breakdown of the different domains of fatigue [16].

Pain Effect Scale (PES): a six-item instrument designed to evaluate the extent to which pain and unpleasant sensations affect various aspects of a patient’s life, including mood, mobility, sleep, work, recreation, and overall enjoyment of life. The scale provides a measure of the severity and impact of pain on daily functioning and well-being. Each item is self-explanatory and scored in a way that higher values reflect a greater negative impact of pain on the patient’s mood and behavior. Total scores range from 6 to 30, offering a straightforward yet comprehensive assessment of pain’s effects on the individual [16].

Sexual Satisfaction Scale (SSS): a four-item scale designed to assess overall sexual adjustment by evaluating satisfaction with physically expressed affection, the variety of sexual activities, and the general quality of the sexual relationship. The items are straightforward, but due to the sensitive nature of the questions, patients may need reassurance regarding the confidentiality of their responses. Scores on the SSS range from 4 to 24, with higher scores indicating greater difficulties or dissatisfaction in sexual aspects of life [16].

Bowel Control Scale (BWCS): a five-item instrument designed to assess bowel control and its impact on an individual’s lifestyle. The scale generates scores ranging from 0 to 26, with higher scores reflecting more significant bowel control issues. Given the sensitive nature of the topic, patients may require reassurance regarding the confidentiality of their responses to ensure accurate and honest reporting [16].

Impact of Visual Impairment Scale (IVIS): a five-item scale that evaluates difficulties with simple visual recognition tasks, such as reading, that cannot be corrected using glasses, contact lenses, or other visual aids. It measures the extent to which vision-related problems affect activities dependent on visual capabilities, excluding issues related to cognitive processing of visual information. Scores range from 0 to 15, with higher values indicating a greater impact of visual impairments on daily life [16].

Perceived Deficits Questionnaire (PDQ): a 20-item self-report tool that evaluates perceived cognitive deficits from the patient’s perspective, offering a broad scope of assessment. It comprises four subscales: Attention/Concentration, Retrospective Memory, Prospective Memory, and Planning/Organization, providing both individual domain scores and a total score. Higher scores reflect greater cognitive impairment, with the total score ranging from 0 to 80. An abbreviated five-item version (PDQ-5) is also available, comprising items most strongly correlated with the total PDQ score and representing all four subscales. The PDQ-5 total score ranges from 0 to 20. While the PDQ-5 is suitable for time-constrained scenarios, the complete 20-item version is recommended for a more detailed evaluation of cognitive functioning [16].

Mental Health Inventory (MHI): an 18-item tool designed to assess overall emotional functioning, encompassing a broad spectrum of both negative and positive emotions. It includes four subscales: Anxiety, Depression, Behavioral Control, and Positive Affect, in addition to a total score. The scores for each subscale and the total range from 0 to 100, with higher scores indicating better mental health. The instrument is self-explanatory and offers a reliable evaluation of emotional well-being while retaining the structure of its subscales. For situations where time is limited, a five-item abbreviated version (MHI-5) is available, which consists of the items most strongly correlated with the full-length summary score. While the MHI-5 is useful for rapid assessments, the full 18-item version is recommended for more comprehensive evaluations, as it is more sensitive to subtle changes in emotional health and distress [16].

Modified Social Support Survey (MSSS): an 18-item self-administered tool designed to assess perceived social support across multiple dimensions, particularly in patients with chronic illnesses. It evaluates four subscales: Tangible Support (material aid or assistance), Emotional/Informational Support, Positive Social Interaction (availability of companions for enjoyable activities), and Affectionate Support. The MSSS provides individual scores for each subscale as well as a total score, all ranging from 0 to 100, with higher scores reflecting greater perceived social support. The instrument focuses on perceived rather than actual support, encompassing various sources without requiring all types of support to come from a single individual. For time-constrained settings, a five-item abbreviated version (MSSS-5) is available, which includes items most strongly correlated with the total score and represents all four subscales. While the MSSS-5 is suitable for rapid assessments, the full 18-item version is preferred for detailed evaluations, as it provides a more comprehensive breakdown of social support dimensions [16].

World Health Organization Quality of Life questionnaire-Bref Version (WHOQOL-BREF): a widely recognized instrument designed to evaluate quality of life across diverse cultural contexts. It comprises 26 items divided into four domains: Physical Health (7 items), Psychological Health (6 items), Social Relationships (3 items), and Environmental Health (8 items). Each domain score is calculated using the mean of the respective items, with higher scores indicating a better quality of life. The Physical Health domain assesses factors such as mobility, daily activities, functional capacity, energy, pain, and sleep. The Psychological Health domain covers aspects like self-image, negative and positive thoughts, self-esteem, mental status, learning ability, memory, concentration, and spirituality. The Social Relationships domain evaluates personal relationships, social support, and satisfaction with sex life. Finally, the Environmental domain focuses on financial resources, living conditions, safety, access to health and social services, opportunities for recreation and skill development, and the physical environment, including noise and pollution. The WHOQOL-BREF is applicable in clinical trials, epidemiological studies, and health policy research, providing a brief yet comprehensive measure of quality of life. It can be self-administered if respondents have adequate reading skills or administered with interviewer assistance when necessary. Scores are scaled positively, meaning higher values correspond to a higher quality of life, making it a valuable tool for cross-cultural research and health service evaluations [17].

Cantril Ladder: A tool used to assess life satisfaction by employing a visual metaphor of a ladder with rungs numbered from 0 to 10. The top rung (10) represents the best possible life satisfaction, while the bottom rung (0) indicates the worst possible life satisfaction. Patients are presented with two diagrams of the ladder during the survey. On the first ladder, participants rate their current level of life satisfaction, reflecting how they feel at that moment. On the second ladder, they are asked to project their expected level of life satisfaction five years into the future. Higher scores on the ladder correspond to greater satisfaction with life. This instrument provides a simple yet effective method to measure both present and anticipated well-being [18].

The study utilized four instruments in both their standard and abbreviated five-item formats: MFIS-5, PDQ-5, MHI-5, and MSSS-5. These shortened versions are scientifically validated and designed to minimize the number of items for participants, especially in scenarios where time constraints are significant. However, it is worth noting that the abbreviated versions may not provide as much detail as their full counterparts [16].

This selection of tools ensures a thorough assessment of various dimensions of health and well-being, providing detailed insights into the physical, psychological, and social aspects of patients’ lives.

### Statistical Analysis

The primary aim was to test for a linear relationship between two continuous variables (dialysis duration in months and questionnaire scores). The Spearman correlation was used to measure the strength and direction of a monotonic relationship between two variables. Spearman correlation was chosen for this analysis due to its ability to detect monotonic relationships without assuming linearity, making it robust against outliers and skewed distributions. Given the wide range of dialysis duration (from 1 to 317 months) and the potential for non-linear relationships, the Spearman correlation provides a more flexible approach compared to the Pearson correlation. If the relationship between dialysis duration and physical fatigue scores is not strictly linear but still follows a monotonic trend (e.g., fatigue tends to increase or decrease consistently with time, but not in a straight line), Spearman correlation would capture this effectively. The *p*-value above the standard significance level of 0.05 was considered a statistically significant monotonic association between the duration of dialysis therapy and the questionnaire scores. The data analysis was performed using IBM SPSS Statistics (Version 27.0, IBM Corp., Armonk, NY, USA).

## 3. Results

Out of 135 dialysis patients screened, 79 (58.5%) were qualified for the study. Table 2 summarizes the demographic, clinical, and dialysis-related characteristics of the study population (n = 79). The cohort consisted of 47 males (59.49%) and 32 females (40.51%), with a mean age of 63.1 years (range: 28–84 years). Regarding marital status, the majority of participants were married (69.62%, n = 55), while 30.38% (n = 24) were single. Educational background varied, with 56.96% (n = 45) having primary education, 26.58% (n = 21) secondary education, and 16.46% (n = 13) university-level education. Most patients (87.34%, n = 69) underwent hemodialysis via an arteriovenous fistula, while 12.66% (n = 10) used a central venous catheter. The mean duration of hemodialysis was 48.1 months (range: 1–317 months). Among the cohort, hypertension was the most prevalent comorbidity, affecting 67.09% (n = 53) of participants, followed by diabetes at 27.75% (n = 22). A prior kidney transplant was reported in 7.59% (n = 6) of patients. Key dialysis-related parameters included a mean Kt/V value of 1.27 (range: 0.34–2.50), a mean ultrafiltration volume of 2168 mL (range: 400–4000 mL), and a mean urea reduction ratio of 0.65 (range: 0.22–0.89).

The study results summarized in Table 1 present the correlations between dialysis duration and the scores across various questionnaires and subscales. Overall, most Spearman correlation coefficients were low, and only a few correlations were statistically significant. For the MFIS, none of the subscales showed significant correlations with dialysis duration, with correlation coefficients (ρ) ranging from −0.167 to −0.064, and *p*-values ranging from 0.154 to 0.589. The PES also demonstrated no significant correlation (ρ = 0.033, *p* = 0.779). In contrast, the SSS revealed a statistically significant positive correlation with dialysis duration (ρ = 0.254, *p* = 0.029), indicating a worsening of satisfaction as dialysis duration lengthened. The BWCS and IVIS showed no significant correlations, with ρ = 0.082 (*p* = 0.489) and ρ = −0.053 (*p* = 0.652), respectively. Regarding the PDQ, neither the total score nor its subscales displayed significant correlations with dialysis duration, with ρ values ranging from −0.208 to −0.067, and *p*-values from 0.072 to 0.572. However, the Planning and Organization subscale demonstrated a near-significant trend (*p* = 0.072), with scores decreasing as dialysis duration increased (ρ = −0.208). For the MHI, no significant correlations were observed across the total score or subscales, with ρ values from −0.082 to −0.005 and *p*-values from 0.487 to 0.968. The MSSS also exhibited no significant correlations with dialysis duration across all subscales, with ρ values from −0.005 to 0.101 and *p*-values ranging from 0.439 to 0.971. The WHOQOL-BREF domains, including Physical, Psychological, Social Relationships, and Environment, did not show significant correlations, with ρ values ranging from −0.074 to 0.186 and *p*-values from 0.124 to 0.665. Lastly, in relation to the Cantril Ladder, no significant correlation was observed between dialysis duration and either current or expected in five years life satisfaction. However, we noted a strong association indicating that patients reporting lower current life satisfaction anticipated a further decline in their life satisfaction over the next five years (ρ = 0.422, *p* = 0.001).

## 4. Discussion

This study examined the relationship between dialysis duration and a range of physical, cognitive, psychosocial, and quality-of-life parameters in patients with end-stage renal disease. An interesting aspect of this study is its focus not only on the parameters that change with the duration of dialysis therapy but also on those that remain unaffected. It might seem intuitive that as dialysis continues, fatigue intensifies, pain worsens, cognitive abilities decline, patients feel less social support, and their quality of life and life satisfaction deteriorate. Vision and overall quality of life might also decline. However, in our study, we did not find a statistically significant effect of dialysis duration on these parameters. Therefore, our discussion will also emphasize these findings.

### 4.1. Sexual Satisfaction

Sexual satisfaction, as measured by the SSS, significantly declines with increasing dialysis duration (*p* = 0.029), underscoring the critical need to address sexual health in the care of dialysis patients [5,19]. Sexual dysfunction in end-stage renal disease stems from a multifactorial interplay of physical, emotional, and relational factors. Physiological contributors include hormonal imbalances, vascular complications, and neurological damage, which lead to symptoms such as diminished libido, erectile dysfunction, and anorgasmia [20]. These challenges are compounded by psychological issues like anxiety, depression, and low self-esteem, further exacerbating the deterioration in sexual satisfaction [5,21]. Despite its prevalence, sexual dysfunction remains underrecognized, with many patients hesitant to discuss their concerns due to stigma or embarrassment, highlighting the necessity for proactive and sensitive communication by healthcare providers [5].

The gender-specific aspects of sexual dysfunction in dialysis patients further emphasize the need for tailored approaches. Women commonly report reduced sexual interest, dyspareunia, and difficulty achieving orgasm, while men frequently experience erectile dysfunction attributed to both physiological and psychological causes [20,22]. Addressing these issues requires a multidisciplinary approach, incorporating hormonal therapies, counseling, and educational programs to mitigate the impact of sexual dysfunction [19]. Additionally, interventions targeting broader relational aspects, such as partner communication and emotional support, are crucial in enhancing outcomes [5]. Successful renal transplantation has also been shown to restore sexual function and satisfaction in some patients, emphasizing the importance of exploring long-term solutions [19].

The significant decline in sexual satisfaction observed in our study aligns with prior findings by Keskin et al. [5] and Filocamo et al. [19]. However, our research uniquely emphasizes the temporal relationship between dialysis duration and sexual satisfaction, suggesting that prolonged treatment exacerbates dissatisfaction. The significant association between dialysis duration and declining sexual satisfaction reinforces the urgency of integrating sexual health into comprehensive dialysis care. Healthcare providers must normalize discussions about sexual health and create a supportive environment for patients to address these concerns. Future research should focus on developing and evaluating targeted strategies to improve sexual health outcomes, ensuring a holistic approach to enhancing the quality of life for dialysis patients, and providing a basis for targeted interventions addressing both physiological and relational aspects.

### 4.2. Cognition

The PDQ results provide a nuanced perspective on the cognitive experiences of patients undergoing dialysis. Although no statistically significant relationship was found between overall PDQ scores and dialysis duration (*p* = 0.076), the Planning and Organization subscale exhibited a trend approaching significance (*p* = 0.072), suggesting a potential vulnerability in executive functions with prolonged dialysis therapy. These findings align with the existing literature, highlighting the multifactorial etiology of cognitive dysfunction in end-stage renal disease, including metabolic imbalances, vascular pathology, and inflammation [6]. Notably, executive dysfunction, encompassing planning, sequencing, and problem-solving, is critical for daily functioning and treatment adherence, making even subtle declines clinically relevant.

The observed stability in overall PDQ scores may reflect adaptive mechanisms or a plateau in cognitive decline over time, consistent with prior findings [8]. However, the near-significant trend in executive functions warrants further attention, as these abilities are essential for managing complex regimens associated with dialysis, such as medication adherence, fluid restrictions, and appointment scheduling. Interestingly, studies suggest that cognitive impairments in dialysis patients do not always correlate directly with dialysis duration or age but are more strongly associated with comorbid conditions like diabetes, hypertension, and cardiovascular disease, as well as inflammatory markers and psychological stress [6,13].

The implications of these findings extend to the broader impact of perceived cognitive deficits on functional outcomes. Patients perceiving cognitive impairments often report limitations in employment, social interactions, and independent living, even when objective deficits are minimal. This underscores the importance of integrating both subjective assessments and neuropsychological evaluations into routine care. Early identification of executive dysfunction and the implementation of targeted interventions, such as cognitive training or compensatory strategies, could mitigate the impact of these impairments and enhance quality of life. Future research should further explore the trajectory of cognitive changes and evaluate multidisciplinary interventions to preserve cognitive health in this vulnerable population. A comprehensive, patient-centered approach will be pivotal in optimizing outcomes for individuals on long-term dialysis.

### 4.3. Life Satisfaction

The Cantril Ladder results offer critical insights into life satisfaction among dialysis patients, emphasizing the nuanced interplay between current well-being and future outlook [18]. Our findings indicated no significant relationship between the duration of dialysis and life satisfaction, whether current or anticipated in five years. However, a strong positive correlation was observed between lower current life satisfaction and poorer anticipated satisfaction in the future (*p* = 0.001), highlighting the profound psychological burden faced by patients with low present satisfaction. This relationship underscores the need for interventions targeting current well-being, as improving present satisfaction could positively influence future expectations and psychological resilience [3,15].

The stability of life satisfaction scores despite prolonged dialysis aligns with previous research suggesting that adaptive mechanisms may buffer the long-term psychological impact of treatment. Patients often develop coping strategies to navigate the challenges of lifestyle restrictions, comorbidities, and emotional distress, stabilizing their sense of satisfaction over time [3,18]. Yet, the significant link between current and future satisfaction points to the importance of addressing present discomforts—physical, emotional, or social—to mitigate declines in anticipated quality of life [13].

The simplicity and utility of the Cantril Ladder make it a valuable tool in clinical practice, providing a snapshot of current life satisfaction while offering predictive insights into future well-being [18]. Identifying patients with low present satisfaction enables healthcare providers to implement tailored interventions, including psychosocial support, pain management, and social integration programs, aimed at improving overall outlook and resilience [3].

Our findings underscore the critical role of non-disease-specific factors, such as environmental and social determinants, in shaping patients’ perceptions of life quality. Future research should focus on these dimensions to develop comprehensive care strategies that enhance both current satisfaction and long-term prognosis [4]. By addressing the multifaceted challenges of dialysis patients, healthcare providers can foster resilience, ensuring improved quality of life both now and in the years to come.

### 4.4. Fatigue

Fatigue is a pervasive and debilitating symptom among dialysis patients, significantly impacting physical, cognitive, and psychosocial functioning, as evidenced by MFIS scores [23,24]. Despite its profound effects, this study found no statistically significant association between dialysis duration and MFIS scores, suggesting that fatigue persists as a chronic challenge irrespective of treatment length. Fatigue in this population often stems from multifactorial causes, including uremia, anemia, electrolyte imbalances, and the physiological strain of the dialysis process itself [7,8]. Commonly reported symptoms include generalized weakness, reduced exercise tolerance, and muscle atrophy, which are further exacerbated by the cyclical nature of fatigue, with symptoms peaking after dialysis sessions [8]. Beyond physical exhaustion, fatigue frequently affects mental health, social engagement, and overall quality of life, limiting patients’ ability to work, maintain relationships, and adhere to treatment regimens [7]. This highlights the multidimensional nature of fatigue and the necessity for holistic management strategies.

The lack of variation in fatigue levels across different durations of dialysis underscores the need for continuous, targeted interventions. Evidence suggests that hybrid exercise programs combining aerobic and resistance training can alleviate post-dialysis fatigue, yet their implementation in clinical practice remains limited [8,23,24]. Non-pharmacological interventions, such as cognitive-behavioral therapy and energy conservation education, alongside medical management of anemia and inflammation, offer promising avenues for comprehensive fatigue management [7]. Given that up to 97% of dialysis patients report fatigue, incorporating routine screening and personalized intervention strategies into standard care is critical. Addressing fatigue not merely as a symptom but as a multidimensional issue can improve life satisfaction and psychosocial outcomes for patients. Future research should explore the long-term efficacy of multidisciplinary fatigue management approaches to optimize care and enhance quality of life for individuals undergoing dialysis.

### 4.5. Pain

Pain is a pervasive and debilitating symptom among dialysis patients, significantly impacting their quality of life and daily functioning. The PES results in this study showed no statistically significant association between dialysis duration and pain levels, suggesting that the burden of pain remains consistent regardless of treatment length. This finding highlights the chronic and persistent nature of pain in end-stage renal disease and underscores the critical need for effective management strategies [9]. Pain in this population has a multifactorial etiology, arising from the dialysis procedure itself, such as dialysis-related headaches linked to rapid shifts in water and electrolytes, as well as comorbidities like osteoarthritis, diabetic neuropathy, and uremic pruritus [9,10]. Additionally, the physiological and psychological toll of end-stage renal disease exacerbates the perception and severity of pain, further complicating its management [9].

Current approaches to pain management in dialysis patients are often inadequate due to the complex interplay between the pharmacokinetics of analgesics, reduced renal clearance, and potential drug interactions unique to this population [9]. This necessitates a balanced and individualized approach that incorporates nonpharmacological interventions such as cognitive-behavioral therapy and physical modalities, alongside cautious pharmacological strategies. The unresolved nature of pain contributes to mood disorders, sleep disturbances, and diminished life satisfaction, emphasizing its role not just as a physical symptom but as a major contributor to psychosocial distress [9]. Addressing pain comprehensively requires integration into the broader scope of dialysis care, with collaborative input from nephrologists, pain specialists, and mental health professionals.

While PES scores did not correlate with dialysis duration, the persistent and multifaceted nature of pain in this population demands ongoing attention and innovation in management. Future research should prioritize the development of multimodal pain relief interventions tailored to the unique needs of dialysis patients, with the ultimate goal of improving their quality of life and overall well-being.

### 4.6. Bowel Control

Bowel dysfunction is a significant yet under-recognized issue among dialysis patients, profoundly affecting their daily lives and quality of life. In this study, the BWCS results revealed no statistically significant association between dialysis duration and bowel dysfunction, highlighting the persistence of these symptoms irrespective of treatment length. This finding underscores the chronic nature of bowel issues in this population and the urgent need for targeted interventions to address these challenges effectively. Functional bowel disorders, such as constipation and diarrhea, are prevalent in end-stage renal disease patients, occurring more frequently in those undergoing dialysis compared to the general population [11]. Contributing factors include strict dietary restrictions, reduced fluid intake, medication side effects, and uremia-induced changes in gut motility and microbial flora. Constipation, in particular, is exacerbated by limited fiber intake due to dietary potassium and phosphate restrictions and compounded by fluid restrictions essential for managing fluid overload [11].

The lack of variation in BWCS scores across dialysis durations suggests that standard management approaches may be insufficient. Many patients face challenges in accessing effective and acceptable treatments, while the stigma surrounding bowel issues often deters them from seeking help. Proactive management strategies should include dietary modifications with dialysis-safe, fiber-rich foods, regular medication reviews to minimize gastrointestinal side effects, and pharmacological interventions like laxatives or stool softeners as needed [11,24]. Encouraging physical activity within patients’ capabilities may further improve bowel function and overall health [24].

Psychosocial factors significantly influence the experience of bowel dysfunction, with embarrassment and stigma leading to emotional distress and reluctance to discuss these issues. Normalizing conversations about bowel health in clinical settings and educating patients on management strategies can empower them to address these challenges effectively. In conclusion, bowel dysfunction remains a persistent issue for dialysis patients, with no significant change in severity over time. Integrating bowel health management into routine dialysis care can enhance physical well-being and alleviate the psychological burden, ultimately improving quality of life. Future research should focus on evaluating tailored interventions and exploring the broader implications of bowel dysfunction on overall health outcomes.

### 4.7. Visual Impairment

Visual impairment is a significant and often disabling issue among dialysis patients, with profound effects on their quality of life. Using the IVIS, this study assessed the extent to which visual problems interfere with daily activities that are not correctable by standard visual aids. Our findings revealed no statistically significant relationship between dialysis duration and IVIS scores, indicating that the burden of visual impairment persists irrespective of the length of time on dialysis. This suggests that visual challenges remain a consistent and ongoing issue for patients, independent of their treatment history.

Visual problems in dialysis patients are closely associated with aging and the complications of chronic kidney disease. Older patients are particularly vulnerable, with visual impairment shown to negatively affect all domains of quality of life, including physical health, psychological well-being, social relationships, and environmental factors [13]. Moreover, diminished vision correlates strongly with reduced life satisfaction, both in current assessments and future projections [13]. These findings highlight the critical need for regular vision screening and targeted interventions to alleviate the impact of vision loss in this population.

Beyond individual well-being, visual impairment is an independent risk factor for adverse health outcomes, such as increased cardiovascular hospitalizations and all-cause mortality in hemodialysis patients [12]. The limitations in performing daily tasks caused by vision problems undermine patients’ independence and pose significant challenges to treatment adherence and overall health maintenance. This necessitates a proactive and integrated approach to managing visual impairment as part of comprehensive dialysis care.

Despite its prevalence, visual impairment remains under-addressed in dialysis patients. Tools like IVIS can help identify those most affected, enabling clinicians to prioritize interventions for these individuals [13]. Strategies may include optimizing the management of contributing conditions like diabetes and hypertension, providing vision rehabilitation services, and offering adaptive devices to enhance functional independence.

While IVIS scores showed no relationship with dialysis duration, the broader evidence underscores the substantial burden of visual impairment in this population. Addressing this challenge requires an integrated approach combining medical management with supportive services to preserve independence and improve quality of life. Future research should investigate tailored interventions for managing visual impairment in dialysis patients, focusing on both clinical and psychosocial outcomes.

### 4.8. Mental Health

The MHI evaluates emotional well-being comprehensively, encompassing aspects such as anxiety, depression, behavioral control, and positive affect. In this study, MHI scores and their subscales showed no statistically significant association with dialysis duration, suggesting that the psychological challenges faced by dialysis patients persist consistently over time. This finding underscores the chronic nature of mental health burdens in this population and highlights the sustained need for mental health support, regardless of treatment duration. Emotional distress, including anxiety, depression, and feelings of social isolation, is a common experience among individuals undergoing dialysis, often exacerbated by the complexities of managing a chronic illness.

Social and family resources are critical for fostering psychological resilience in these patients, as evidenced by the role of emotional and material support from caregivers, healthcare providers, and social networks in mitigating the psychological burden of chronic illnesses [14]. The stable MHI scores observed over time may reflect the resilience of some patients or the buffering effects of social support. However, disparities in access to such resources could lead to variable psychological outcomes, emphasizing the importance of individualized interventions to address specific patient needs.

The high prevalence of anxiety and depression among dialysis patients, documented in prior studies, further underscores the need for proactive screening and mental health interventions [14]. Tailored approaches, such as cognitive-behavioral therapy, peer support programs, and educational initiatives, could significantly enhance emotional well-being and quality of life. While these results indicate stability in psychological health over time, they should not be interpreted as an absence of need. Instead, they highlight the importance of integrating mental health care into routine dialysis management to prevent further deterioration and improve long-term outcomes.

The findings on MHI scores emphasize the critical need for sustained and accessible mental health support for dialysis patients. Comprehensive care models that address both medical and psychological aspects are essential for fostering resilience and improving overall patient outcomes. Future research should focus on identifying and implementing the most effective mental health interventions within dialysis settings, prioritizing patient-centered and equitable care strategies.

### 4.9. Social Support

The MSSS provides critical insights into the role of perceived social support in the lives of patients undergoing chronic dialysis [16]. Social support, encompassing emotional and tangible assistance, is a well-established determinant of psychosocial well-being and health outcomes in the management of chronic illnesses. Our study found no statistically significant changes in MSSS scores or their subscales related to dialysis duration, suggesting that the perception of social support remains stable over time. This stability implies that the presence or absence of supportive networks is not inherently influenced by the length of time a patient has been on dialysis treatment.

Social interaction is a cornerstone of well-being and significantly impacts health outcomes in individuals with end-stage renal disease [25,26]. Chronic illness often limits access to social support, which can exacerbate both emotional and physical health challenges [1]. For dialysis patients, fostering strong social networks is crucial to mitigating the psychological and social isolation commonly associated with long-term treatment. The consistent MSSS scores observed in this study underscore the need to ensure reliable social resources throughout the patient’s treatment journey.

Higher levels of perceived social support have been strongly linked to better survival rates and quality of life in patients with chronic conditions, including cancer and cardiovascular disease [15]. Social support buffers stress and promotes adherence to treatment regimens, which is especially critical for dialysis patients. Healthcare providers should prioritize interventions to enhance social support as a modifiable factor for improving outcomes. Tailored programs addressing the unique needs of dialysis patients could improve their perception of support, positively influencing both health and longevity [15,25].

The observed stability in MSSS scores may reflect the resilience of existing social networks or the inherent challenges dialysis patients face in establishing new ones. Research shows that patients with robust perceived support report better mental health-related quality of life, while those with limited support or high interpersonal sensitivity face worse outcomes over time [1]. These findings emphasize the importance of integrating social support strategies into standard dialysis care.

In conclusion, the MSSS results highlight social support as a stable yet essential component of comprehensive dialysis care. Although dialysis duration does not appear to influence perceived support, its importance in managing chronic kidney disease cannot be understated. Sustaining and enhancing social support networks for long-term dialysis patients is vital for improving quality of life and treatment adherence. Future research should explore targeted interventions to strengthen social resources and assess their impact on both psychological resilience and physical health outcomes.

### 4.10. Quality of Life

The WHOQOL-BREF results provide critical insights into the multidimensional quality of life in dialysis patients, revealing no significant association between dialysis duration and the scores across its four domains: Physical Health, Psychological Health, Social Relationships, and Environmental [17]. This stability suggests that while dialysis significantly impacts patients’ lives, the passage of time alone does not necessarily exacerbate the perceived decline in quality of life, potentially reflecting patients’ psychological and social adaptation to their condition and treatment [3]. However, quality of life in end-stage renal disease patients remains substantially lower compared to the general population, with older age, high comorbidity, and low serum albumin levels identified as key contributors to poorer outcomes [4]. These findings emphasize the need for targeted interventions that address specific quality of life components rather than assuming uniform deterioration.

The Physical Health domain, often significantly impaired in dialysis patients, did not show a worsening trend with increased treatment duration, likely due to advancements in dialysis techniques and supportive care. Nonetheless, poor physical health shortly after dialysis initiation remains a predictor of mortality, highlighting the necessity for early and sustained intervention [3,4,19]. Psychological well-being, while stable in this study, remains a critical concern, as patients’ perceptions of their disease and treatment profoundly influence health-related quality of life. Social support and psychological resilience play crucial roles in maintaining this stability, underscoring the importance of ongoing mental health support [1,3,14].

The Social Relationships domain reflects the impact of dialysis on personal connections, with stable scores suggesting that many patients develop strategies to preserve their social networks. However, barriers to social engagement must be addressed, as robust social support predicts improved survival and health outcomes in this population [15]. Similarly, the Environmental domain, encompassing financial and systemic factors, remains unchanged, signaling the need for policies enhancing healthcare access and financial support to alleviate external stressors impacting the quality of life [3].

In conclusion, the WHOQOL-BREF results highlight the complexity of factors influencing the quality of life in dialysis patients. Although dialysis duration does not directly affect quality of life scores, these findings underscore the persistent need for interventions tailored to each quality of life domain. Future research should prioritize identifying modifiable determinants and developing strategies to enhance the holistic well-being of this vulnerable population.

### 4.11. Summary of Discussion

Our study highlights that certain parameters, such as fatigue, mental health, and quality of life, remain stable over time in patients undergoing long-term dialysis. Several factors may explain this stability. First, patients may develop coping mechanisms that mitigate the impact of chronic challenges. Second, advancements in dialysis technology and healthcare interventions likely contribute to these outcomes. Third, the study population may include individuals who are inherently more resilient and better able to adapt to long-term dialysis. Finally, the assessment tools used may lack the sensitivity to detect gradual, long-term changes in these domains.

These findings align with prior research. For instance, Manfredini et al. [23] and Zuo et al. [7] reported that fatigue persists among dialysis patients regardless of treatment duration, influenced by complex physiological and psychological factors. Similarly, studies by Wang et al. [14] and Sousa et al. [26] suggest that stable mental health scores over time may result from patients’ social and emotional adaptation. Furthermore, our results on quality of life are consistent with those by de Rooij et al. [3], who observed that while dialysis patients have a consistently lower quality of life compared to the general population, these scores do not worsen over time.

Our study builds on this understanding by providing a multidimensional analysis of quality of life. Unlike prior research, such as that by de Rooij et al. [3], which focused on isolated metrics, our findings emphasize the need for tailored strategies that address both medical and social determinants of health.

However, our findings differ from studies by Palmer et al. [27,28] and Tsai et al. [29], which identified a strong correlation between dialysis duration, depression, and adverse outcomes. These discrepancies may be due to differences in measurement tools and population characteristics. Unlike prior meta-analyses that relied on depression-specific instruments, our study used broader quality-of-life measures (WHOQOL-BREF, MHI). Additionally, our sample was limited to Polish patients from a single center, potentially influenced by cultural or healthcare system factors. These findings underscore the importance of considering individual and contextual factors when evaluating psychosocial outcomes in dialysis patients.

Our findings also have practical implications for clinical management. The observed decline in sexual satisfaction over time underscores the need for integrating sexual health assessments and interventions into routine care. This includes fostering a safe environment for discussing sensitive topics and offering multidisciplinary support, such as counseling and hormonal therapies.

The stability of fatigue, pain, and mental health scores highlights the importance of consistent management strategies for these chronic issues. Personalized exercise programs and cognitive-behavioral therapy may mitigate fatigue, while multimodal pain management approaches can address persistent pain symptoms.

Lastly, the findings on life satisfaction suggest that enhancing patients’ current well-being can positively influence their future outlook. Psychosocial interventions, such as support groups or resilience training, should be prioritized to improve patients’ quality of life and coping mechanisms. These insights pave the way for a more holistic approach to the care of long-term dialysis patients.

## 5. Limitations

This study has several limitations that should be acknowledged to provide context for the interpretation of the findings. One key limitation is its single-center design, which inherently restricted the size of the study group. The relatively small sample size may have reduced the ability to achieve statistical significance in some analyses. The cross-sectional design of our study limits the ability to infer causality or detect longitudinal changes in quality of life measures. Nonetheless, the observed trends and relationships identified in this study offer valuable insights and serve as a foundation for future research involving larger, more diverse populations.

Another limitation lies in the reliance on patient self-reports collected through standardized questionnaires. While these tools are well-validated and offer a practical means of assessing patient experiences, they do not substitute for objective evaluations conducted by medical specialists such as ophthalmologists, psychiatrists, or psychologists. Consequently, the findings may be influenced by subjective biases or individual variations in self-perception, which could affect the accuracy of the results. Future studies incorporating a combination of self-reported data and objective clinical assessments would provide a more comprehensive understanding of the parameters examined.

One notable limitation of our findings is the generally low correlation coefficients observed for most parameters, despite some achieving statistical significance. The low correlations highlight the multifactorial nature of the health challenges faced by dialysis patients. These results underscore the importance of interpreting statistical significance alongside the strength of the observed correlations, particularly in a complex, multidimensional population.

These limitations highlight the need for caution when generalizing the findings to broader populations or interpreting causality. Despite these challenges, the study contributes meaningful insights into the relationships between dialysis duration and various health-related parameters, emphasizing the importance of ongoing research in this area.

## 6. Novelty and Implications for Future Research

This study stands out due to its multidimensional approach, integrating physical, cognitive, and psychosocial domains to provide a comprehensive understanding of the patient experience. While many of our findings align with existing literature, such as the stability of fatigue and pain levels over time, we uniquely identify vulnerable areas like sexual satisfaction and cognitive planning. Moreover, the strong correlation between current and anticipated life satisfaction underscores the importance of addressing psychological resilience in dialysis care. These findings open new avenues for research, particularly in designing interventions that are not only disease-specific but also patient-centered, addressing the nuanced interplay of medical, psychological, and social factors.

## 7. Summary of Findings and Conclusions

This study explored the impact of dialysis duration on various aspects of patient health and well-being. The findings reveal that the duration of dialysis significantly influences sexual satisfaction, with prolonged dialysis associated with a notable deterioration in this domain. Furthermore, a trend suggesting negative effects on cognitive function, specifically in planning and organization, was observed, though it did not reach statistical significance. Additionally, patients who currently perceive their quality of life as poor expressed a significant expectation of further decline in the future.

Importantly, no relationship was identified between the duration of dialysis and several other health-related parameters. Fatigue, as assessed by the MFIS test, pain (PES test), bowel control (BWCS test), vision (IVIS test), mental health (MHI test), social support (MSSS test), quality of life (WHOQOL-Bref test), and life satisfaction (Cantril Ladder) were not significantly influenced by the length of time on dialysis. These findings suggest a complex and multifaceted nature of health outcomes in dialysis patients, where certain areas remain resilient to the effects of prolonged therapy, while others are more vulnerable.

The results underscore the need for targeted, individualized interventions to address the most vulnerable domains, particularly sexual satisfaction, cognitive function, and psychological health. Clinicians should prioritize addressing sexual health, providing consistent support for chronic symptoms like fatigue and pain, and promoting psychological resilience through targeted interventions. These strategies can collectively enhance patients’ quality of life, even in the absence of significant changes in other domains.

While this study highlights the stability of certain health-related outcomes over the duration of dialysis, it is essential to interpret these findings in the context of the study’s design and population. Factors beyond dialysis duration, such as individual resilience and comorbidities, may play a significant role in shaping quality of life and mental health in this population.

By integrating these findings into clinical practice, healthcare providers can adopt a more holistic approach to patient care, focusing on the nuanced and multidimensional challenges faced by dialysis patients. Future research involving larger, multicenter cohorts, as well as objective clinical evaluations, is essential to confirm these findings and guide the development of comprehensive therapeutic strategies. Additionally, further exploration and validation of interventions aimed at improving specific aspects of health and well-being are necessary to enhance the overall quality of life for long-term dialysis patients.

## Figures and Tables

**Table 1:** Impact of dialysis duration on fatigue, pain, sexual satisfaction, bowel control, vision, cognitive deficits, mental health, social support, quality of life, and life satisfaction.

Questionnaire	Subscale	Number of Items	Scoring Range	Spearman Correlation Coefficient (ρ)	*p*-Value
Modified Fatigue Impact Scale (MFIS)	MFIS total score	21	0–84	−0.093	0.431
	MFIS-5 score	5	0–20	−0.108	0.361
	Physical subscale	11	0–36	−0.080	0.497
	Cognitive subscale	10	0–40	−0.064	0.589
	Psychosocial subscale	2	0–8	−0.167	0.154
Pain Effects Scale (PES)		6	6–30	0.033	0.779
Sexual Satisfaction Scale (SSS)		4	4–24	0.254	0.029
Bowel Control Scale (BWCS)		5	0–26	0.082	0.489
Impact of Visual Impairment Scale (IVIS)		5	0–15	−0.053	0.652
Perceived Deficits Questionnaire (PDQ)	PDQ total score	20	0–80	−0.162	0.168
	PDQ-5 score	5	0–20	−0.160	0.172
	Attention/Concentration subscale	5	0–20	−0.167	0.156
	Retrospective Memory subscale	5	0–20	−0.166	0.159
	Prospective Memory subscale	5	0–20	−0.067	0.572
	Planning/Organization subscale	5	0–20	−0.208	0.072
Mental Health Inventory (MHI)	MHI total score	18	0–100	−0.005	0.968
	MHI-5 score	5	0–100	−0.082	0.487
	Anxiety Subscale	5	0–100	−0.042	0.723
	Depression Subscale	5	0–100	−0.007	0.952
	Behavior Control Subscale	4	0–100	−0.008	0.947
	Positive Affect Subscale	4	0–100	−0.013	0.914
Modified Social Support Survey (MSSS)	MSSS total score	18	0–100	−0.005	0.971
	MSSS-5 score	5	0–100	0.012	0.928
	Tangible Support Subscale	4	0–100	0.006	0.963
	Emotional/Informational Support Subscale	8	0–100	0.101	0.439
	Affectionate Support Subscale	3	0–100	0.097	0.443
	Positive Social Interaction Subscale	3	0–100	0.087	0.511
The World Health Organization Quality of Life questionnaire-Brief Version (WHOQOL-BREF)	Physical domain	7	4–20	0.101	0.404
	Psychological domain	6	4–20	−0.074	0.540
	Social Relationships domain	3	4–20	−0.054	0.665
	Environment domain	8	4–20	0.186	0.124
Cantrill ladder	Present satisfaction with life	1	0–10	0.066	0.623
	Expected in 5 years satisfaction with life	1	0–10	0.051	0.704
	Expected vs. present satisfaction with life			0.422	0.001

MFIS—Modified Fatigue Impact Scale, PES—Pain Effect Scale, SSS—Sexual Satisfaction Scale, BWCS—Bowel Control Scale, IVIS—Impact of Visual Impairment Scale, PDQ—Perceived Deficits Questionnaire, MHI—Mental Health Inventory, MSSS—Modified Social Support Survey, WHOQOL-BREF—the World Health Organization Quality of Life questionnaire-Brief Version.

**Table 2:** Demographic, clinical, and dialysis-related characteristics of the study population.

		n/M [Range/%] (SD)
sex	male	47 [59.49%]
	female	32 [40.51%]
age		63.1 years [28–84] (12.2)
marital status	single	24 [30.38%]
	married	55 [69.62%]
education	primary	45 [56.96%]
	secondary	21 [26.58%]
	university	13 [16.46%]
vascular access for hemodialysis	arteriovenous fistula	69 [87.34%]
	central venous catheter	10 [12.66%]
duration of hemodialysis		48.1 months [1–317] (62.3)
comorbidities	diabetes	22 [27.75%]
	hypertension	53 [67.09%]
kidney transplant in the past		6 [7.59%]
Kt/V		1.27 [0.34–2.50] (0.31)
ultrafiltration		2168 mL [400–4000] (950)
urea reduction ratio		0.65 [0.22–0.89] (0.09)

n—number; M—mean; SD—standard deviation.

## Data Availability

The data presented in this study are available on request from the corresponding author. The data are not publicly available due to privacy restrictions.
